# A Psychometric Evaluation of the Danish Version of the Theory of Mind Storybook for 8–14 Year-Old Children

**DOI:** 10.3389/fpsyg.2016.00330

**Published:** 2016-03-08

**Authors:** Lars Clemmensen, Agna A. Bartels-Velthuis, Rókur av F. Jespersen, Jim van Os, Els M. A. Blijd-Hoogewys, Lise Ankerstrøm, Mette Væver, Peter F. Daniel, Marjan Drukker, Pia Jeppesen, Jens R. M. Jepsen

**Affiliations:** ^1^Child and Adolescent Mental Health Centre, Mental Health ServicesGlostrup, Denmark; ^2^Faculty of Health Science, University of CopenhagenCopenhagen, Denmark; ^3^University of Groningen, University Medical Center Groningen, University Center for PsychiatryGroningen, Netherlands; ^4^King’s Health Partners, Department of Psychosis Studies, Institute of Psychiatry, King’s College LondonLondon, UK; ^5^Department of Psychiatry and Psychology, Maastricht University Medical CentreMaastricht, Netherlands; ^6^INTER-PSYGroningen, Netherlands; ^7^Department of Psychology, University of CopenhagenCopenhagen, Denmark; ^8^Department of Child and Adolescent Psychiatry, Zealand RegionNæstved, Denmark; ^9^Lundbeck Foundation Center for Clinical Intervention and Neuropsychiatric Schizophrenia ResearchCopenhagen, Denmark; ^10^Center for Neuropsychiatric Schizophrenia Research, Psychiatric Centre GlostrupGlostrup, Denmark

**Keywords:** Theory-of-Mind, validation, autism spectrum disorders

## Abstract

**Background:** Theory-of-Mind (ToM) keeps on developing in late childhood and early adolescence, and the study of ToM development later in childhood had to await the development of sufficiently sensitive tests challenging more mature children. The current study aimed to investigate the psychometric properties of the Danish version of the Theory-of-Mind Storybook Frederik (ToM-Frederik).

**Methods:** We assessed whether ToM-Frederik scores differed between a group of 41 typically developing (TD) children and a group of 33 children with High Functioning Autism Spectrum Disorder (HFASD). A lower mean ToM-Frederik score was expected in the HFASD group. To determine the convergent validity of ToM-Frederik, potential associations with Strange Stories and Animated Triangles (AT) were analyzed. Furthermore, potential associations between ToM-Frederik and the Social Responsiveness Scale (SRS) and between ToM-Frederik and the Social Emotional Evaluation (SEE) Total score were analyzed.

**Results:** A significantly higher ToM-Frederik score was observed in the TD group compared to the HFASD group. Furthermore, the convergent validity of ToM-Frederik as a measure of ToM was supported by significant and positive associations with the Strange Stories and the AT scores in the HFASD group, whereas ToM-Frederik was significantly correlated with Strange Stories, but not with AT in the TD group. ToM-Frederik was not significantly associated with SRS in neither the HFASD nor the TD group.

**Conclusion:** The findings are supportive of ToM-Frederik as a valid indicator of deficits at the group level in children with HFASD between 7 and 14 years of age. Furthermore, the convergent validity is supported.

## Introduction

In order to study the interplay between social cognition and psychopathology in the pathogenesis of mental disorders we need more sensitive and specific measures of the various aspects of social cognition including different levels of Theory-of-Mind.

Theory-of-Mind (ToM) is the ability to infer intentions, desires and beliefs (Frith and Frith, 2012). Deficits in this ability are present in a wide range of mental disorders, such as autism spectrum disorders (ASD) and other neurodevelopmental disorders, personality disorders, other non-psychotic disorders, schizophrenia, and affective disorders (Brune and Brune-Cohrs, 2006).

The ‘gold standard test’ of comprehending other persons’ minds is to grasp that others can hold false beliefs (FB) different from one’s own (correct) knowledge (Brune and Brune-Cohrs, 2006). The original and still prevailing ‘litmus’ tests for this ability are (first-order) FB tasks (Abell et al., 2000), such as the Sally and Anne test (Wimmer and Perner, 1983). FB reasoning has been shown to be detectable as early as infancy (Baillargeon et al., 2010), but the general perception is that typically developing (TD) children will attain this ability at the age of 4, through a progression of stages starting at 9 months with early aspects of joint attention (Paparella et al., 2011). A developmental milestone has been observed at around the age of 18 months, when children become aware that their own mental states are distinct from others’ (Korkmaz, 2011). At the age of 4 there is still considerable instability in the understanding of FB (Hughes et al., 2005), but from the age of 4–6 this understanding becomes firmly established (Brune and Brune-Cohrs, 2006; Blakemore, 2008; Jha and Singh, 2009).

Still, ToM comprises far more than FB alone, like for instance the understanding of desires and emotions of others (Blijd-Hoogewys et al., 2008). Testing ToM with only FB tasks leaves out more sophisticated social-cognitive capacities such as understanding metaphor, irony and ‘faux pas’ (a socially awkward or tactless act) (Brune and Brune-Cohrs, 2006). Also, first-order FB comprehension might be mastered at younger ages, but second-order FB comprehension will not be mastered before the age of 6–7 years in TD children (Dumontheil et al., 2010). Second-order FB, where the child must think about a second individual’s thoughts about a third individual’s thoughts about an event (meta-representation), is often assessed through the ‘Ice-cream man’ task (Sullivan et al., 1994). Lies and jokes (Happé, 1994) and ‘faux pas’ may not be reliably understood by TD children before the age of 8–11 years (Brune and Brune-Cohrs, 2006), indicating that ToM further improves in late childhood and early adolescence (Dumontheil et al., 2010). Although ToM seems to continue to develop after the age of 6–7 years, Dumontheil et al. (2010) found little evidence to support this assumption. Since most standard ToM tasks are passed by the age of 5 years, ceiling effects are presumably obscuring the observation of any further development, as these tests do not measure more complex abilities (White et al., 2009; Dumontheil et al., 2010). Therefore, more comprehensive and challenging ToM tests, such as the Strange Stories task, have been developed (Happé, 1994; Brent et al., 2004).

Among the most widely used advanced ToM tasks are the (*revised) Strange Stories task* (Happé, 1994; White et al., 2009). The Strange Stories include tasks on pretense, joke, lie, white lie, misunderstanding, persuasion, appearance/reality, figure of speech, irony, double bluff, contrary emotions, and forgetting. The task focuses on participants’ understanding of belief but is developmentally appropriate for older children because of the additional demands it places on using this understanding of beliefs in complex, contextualized scenarios (Devine and Hughes, 2013). The Strange Stories task has been widely used in studies of clinical populations and, more recently, in studies of TD school-aged children (e.g., Ronald et al., 2006; Lecce et al., 2010). It has proven successful in numerous studies in older children, showing that children, adolescents and even adults with High Functioning Autism Spectrum Disorder (HFASD) perform significantly worse on the Strange Stories task than TD individuals (Happé, 1994; White et al., 2009). Group differences between samples of TD and HFASD children and adolescents have also been shown with the Danish version of the original Strange Stories task (Kaland et al., 2008).

Children and adults with ASD and with an intelligence quotient (IQ) greater than 70 (HFASD) (Jacobs and Richdale, 2013), typically pass standard tests of first-order ToM such as FB tasks, although they are delayed in acquiring this understanding (Brent et al., 2004). The majority of individuals with HFASD also pass second-order FB tests (Kaland et al., 2008), but might not do so before their teens (Korkmaz, 2011). However, difficulties passing the tests increase with test complexity. As an example, the study by Kaland et al. (2008) found that children and adolescents with high-functioning autism or Asperger’s syndrome pass standard ToM tasks but showed significant impairments in the more ‘advanced’ ToM tests with complex social contexts.

A more recent, advanced ToM task is the *Animated Triangles (AT) test* (Abell et al., 2000). This task consists of series of computer-presented animations, and measure both the understanding of the events depicted in the animations (Appropriateness) and the degree of intentional attribution (Intentionality) (Salter et al., 2008). This test has successfully been used in a growing number of experiments to investigate ToM and agency attribution in individuals with and without ASD (White et al., 2011). Studies in both children and adolescents (Abell et al., 2000; Salter et al., 2008; Schwenck et al., 2012) and adults (Castelli et al., 2002; White et al., 2011) have found lower Appropriateness score in ASD compared to TD. However, whereas differences in Intentionality have been found in adults (White et al., 2011), this is often not the case in samples of children and adolescents (Abell et al., 2000; Salter et al., 2008). Nevertheless, this is considered a promising way to investigate the more subtle differences in ToM abilities in HFASD children and adolescents (Salter et al., 2008).

In the present study a new advanced ToM task was explored. The *Theory-of-Mind Storybook Frederik* (ToM-Frederik – (Blijd-Hoogewys and Bartels-Velthuis, 2007), which was developed for children aged 10–12, contains ToM tasks of different levels of difficulty, including first-order FB, deception, second-order FB, white lie, irony, double bluff and ‘faux-pas’ (Bartels-Velthuis et al., 2011). This task comprises both yes/no and justification questions and a scoring system that allows for a detailed categorization of the answers in potentially different types of ToM deficits, e.g., the exaggerated type of ToM alteration, termed HyperToM (Clemmensen et al., 2014). This makes ToM-Frederik a relevant instrument for studies addressing the hypothesis that specific types of ToM deficits may be involved in the development of specific symptoms (Shamay-Tsoory et al., 2007; Montag et al., 2011).

The overall aim of the present study was to examine aspects of validity and reliability of the Danish version of ToM-Frederik. This version (ToM-Frederik) has been applied in a population study of 1600 Danish 11–12-year-old children (Clemmensen et al., 2014), but has not been applied to any clinical populations. The original Dutch Theory of Mind Storybook – Frank has been assessed in TD children and children with PDD-NOS and found to have good psychometric qualities (good internal consistency, test–retest reliability, inter-rater reliability, construct validity, and convergent validity (Blijd-Hoogewys et al., 2008).

We included a group of TD children and a group of children with HFASD aged 7–14. The rationale for including a group with ASD was that the presence of ToM deficits in this group is well-established in both children and adults (Yirmiya et al., 1998; Baron-Cohen, 2001; Colle et al., 2007).

First, we wanted to explore if ToM-Frederik could detect the expected ToM deficits in the HFASD group as reflected in significant differences in mean scores between the HFASD and the TD groups. Second, we wanted to explore the converging validity as reflected in potential significant associations between scores derived from ToM-Frederik and the Strange Stories, which we consider to be the gold standard ToM Task, and also AT. Third, to test if ToM is associated with social function, we wanted to assess potential associations between ToM-Frederik and (1) the Social Emotional Evaluation (SEE) test and the SEE parental questionnaire (SEQ) although the SEE only covers the age range 6–12 and (2) the Social Responsiveness Scale (SRS). SEE and SEQ evaluate the social skills of the child (Wigg, 2013) and SRS covers various aspects of interpersonal communication, reciprocal behavior, and repetitive/stereotypic behavior. The inclusion of these measures was based on the commonly accepted idea that social cognitive deficits affect social function and skills. Finally, we wanted to examine the test–retest reliability, and a possible test–retest effect, of the ToM-Frederik.

## Materials and Methods

### Autism Spectrum Disorder Group

The sample consisted of children diagnosed with ASD at the Child and Adolescent Psychiatric Centre in the Zealand Region of Denmark or at the Child and Adolescent Mental Health Centre, Mental Health Services in the Capital Region of Denmark. The children were recruited during the study period from August 2013 until September 2014. The inclusion criteria were as follows: (1) age 7–14, (2) a diagnosis of ASD according to the ICD-10 criteria (DF 84.0–84.9), (3) the diagnosis is based on a formal evaluation by a specialist in child and adolescent psychiatry following the Danish, national clinical guidelines for assessment and diagnosis of ASD, (4) the absence of an intelligence level below the normal range, as reflected in the absence of a mental retardation diagnosis (DF 70.x), and an IQ at >70 measured by a full WISC-III or WISC-IV or by a scale score of four or above in both the Vocabulary and Block Design subtests from the WISC-III or WISC-IV (if full test not administered), (5) sufficient Danish language skills to participate in the ToM tasks, and (6) parental written informed consent. As ToM-Frederik is primarily aimed at children aged 10–12, the majority of the children were recruited within in this age-range but we also included younger and older children to assess if the age range of the task could be extended. Originally, 37 participants with ASD were included but four participants were excluded as they did not fulfill the criteria regarding intelligence level. Thus, a total of 33 children with HFASD were included: 3 children aged 7–8, 11 children aged 9–10, 12 children aged 11–12, and 7 children aged 13–14.

### Typically Developing Group

The names and addresses of a random sample of children aged 7–14 years were extracted from the Civil Registration System (Det Centrale Personregister; CPR). The TD children were invited to participate in the study by mail in a neutral white envelope, addressed to the child’s parents. The parents were asked to sign up (through an online booking system) for an assessment of their child at the clinic. Inclusion criteria were as described above except no parental report of (1) developmental disorders, or (2) special educational needs. Originally, 43 participants were included but two participants were excluded due to not fulfilling the criteria for intelligence level. We included a total of 41 TD children: 8 children aged 7–8, 13 children aged 9–10, 14 children aged 11–12, and 6 children aged 13–14.

### Procedure

The HFASD group was tested at the clinic by clinically experienced staff who received training and supervision in administration of the tests. Trained researchers also tested the TD group, either at home or at the clinic as preferred by the parents. To avoid potential systematic fatigue effects on the three ToM-tasks and SEE test performance, the order of the administration changed successively for each new participant within both groups (first participant; 1;2;3,4; second participant; 2;3;4;1; third participant; 3;4;1;2; etc.).

### Ethics

The study was approved by The Danish Data Protection Agency (J.nr 2013-011-02230) in the Capital Region of Denmark (J.nr. 2007-58-0015) and The National Committee on Health Research Ethics was consulted (H-2-2013-FSP22) in accordance with national guidelines.

### Instruments

The ToM Storybook Frederik (ToM-Frederik) is the Danish version of the ToM Storybook Frank (Blijd-Hoogewys and Bartels-Velthuis, 2007), which tests the understanding of first-order FB, deception, second-order FB, white lie, irony, double bluff and ‘faux pas.’ Children are presented with 16 pictures while listening to a tape recording of the storybook read aloud by a professional actor. Children are asked a total of 16 ‘test’ and 8 ‘justification’ questions, covering a range of ToM abilities. The 16 ‘test’ answers are scored 1 (for a correct understanding of the situation) or 0 (for an incorrect answer). The range of the ‘test’ sum score is 0–16. The justifications questions (such as “Why did the mother say that?”,“Why does Frederik think that?”) are classified according to 23 predefined categories and scored on an ordinal scale (predefined for each situation). Categories include Desire: The answer refers to the protagonist’s desire with respect to the situation. It involves wanting or desiring something; Fact belief: The child refers to the protagonist’s knowledge. It involves thinking, knowing, being sure of, expecting or recognizing; Situational – Dwelling on the situation without reference to the mental state of the protagonist. Scores depend on the level and quality of references made to the thoughts, beliefs, feelings or intentions of the story characters or the child itself. The range of the ‘justification’ sum score is 0–20. The test and justification scores are summed in a total score of ToM skills (range 0–36, with a higher score indicating better performance) that serves as the primary measure (Clemmensen et al., 2014). Ratings for ToM-Frederik were carried out by LC and RJ. Based on all 74 participants the inter-rater reliability was excellent (*r* = 0.96, *p* < 0.001) (George and Mallery, 2003). The typical administration time of the ToM-Frederik was 15 min.

To measure the test–retest reliability and a possible test–retest effect of the ToM-Frederik, we administered this test for a second time to the 11–12-year-old children in the TD group 2–3 weeks after the first administration.

The *Strange Stories* test assesses the child’s understanding of: pretense, joke, lie, white lie, misunderstanding, persuasion, appearance/reality, figure of speech, irony, double bluff, contrary emotions, and forgetting (Happé, 1994) and provides means for testing advanced ToM-ability, suitable for TD as well as for both children (Brent et al., 2004) and adults with HFASD (Sanders, 2009). Short vignettes are read aloud by the interviewer, whereupon subjects are asked to explain why a character says something that is not literally true (White et al., 2009). We used a Danish translation of the revised version of the Strange Stories (White et al., 2009) and included the eight Mental State Stories (awarded 0, 1, or 2 points per story, the range of the sum score is 0–16) and the eight new Natural Physical State Stories (awarded 0, 1, or 2 points per story, the range of the sum score 0–16). The mental and physical state sets both requires the integration of information between sentences and inference from implicit information, but only the mental state set requires mentalizing (White et al., 2009). Thus, adjusting the Strange Stories Mental State score for the Strange Stories Physical State score provides a more focused measure of the ability to reason about mental states specifically. Ratings for the Strange Stories were carried out by LC and RJ. Based on independent ratings of the responses of 20 randomly selected participants, the inter-rater reliability for the Mental State Stories was good (*r* = 0.73, *p* < 0.001) and the Natural Psychical State Stories (*r* = 0.92, *p* < 0.001) was excellent (George and Mallery, 2003). All ratings were compared to the ratings of a senior researcher (JJ). No interrater-reliability with the senior researcher was calculated, as consensus scores were determined via discussion in all cases of differences in ratings.

The AT (Abell et al., 2000) task consists of a series of computer-presented animations. These animations show one large red and one small blue triangle moving around the screen (White et al., 2011). The participants are instructed that, while watching the animations, they have to give a concurrent verbal description of what they think is happening. The participants were expected to evaluate and characterize the interplay between the triangles or lack hereof. These animations span scenarios from ‘Random’ to ‘Goal-Directed’ and ‘ToM’-type situations, and performance in terms of verbal descriptions were rated on scales for Appropriateness and Intentionality. The AT Appropriateness score measures the understanding of the event depicted in the animations, as intended by the designers (0–3, with 3 being a clear precise answer). A total score ranging from 0 to 12 was calculated for each type of animation (see Castelli et al., 2000 for further details). The Intentionality score reflects the use of mental state terms, with scores ranging from 0 (non-deliberate action) to 5 (deliberate action aimed at affecting another’s mental state). Total AT Intentionality score was calculated as the mean for each animation type. If the participant failed to produce a verbal description, AT Appropriateness was rated 0 and AT Intentionality as the mean of the other answers for that type of animations. Failure to produce verbal descriptions for two or more animations within a given category meant exclusion from this particular analysis. Separate scores were calculated for the Random, Goal-Directed and ToM sets of animations. Ratings were carried out by LC and RJ. Based on 20 randomly selected participants, the inter-rater reliability for both the AT Appropriateness score (*r* = 0.824, *p* < 0.001) and the AT Intentionality score (*r* = 0.894, *p* < 0.001) were excellent (George and Mallery, 2003). These ratings were compared to the ratings of a senior researcher (JJ). No interrater-reliability with the senior researcher was calculated, as consensus scores were determined via discussion in all cases of difference in ratings.

We also applied an objective method, originally developed for adults, using multiple choice questions (MCQ) (White et al., 2011). At the end of each animation participants would be asked to categorize it as one of three types: ‘no interaction’ (Random), ‘physical interaction’ (Goal Directed), and ‘mental interaction’ (ToM) (referred to hereafter as AT MCQ-categorization score: 0–12 points). In cases of ToM type animations they were asked additional questions to test their understanding of the mental states depicted in the animation. The participants could choose between one of five listed adjectives to best match the feelings of each of the triangles at the end of the animation; a separate list was provided for each triangle in each animation (referred to hereafter as MCQ-feelings score: 0–8 points).

Performances on the *Block Design* and the *Vocabulary* subtests of *WISC-III* or *WISC-IV* were used as indices of the intelligence level. These subtest scores are known to be highly correlated with IQ on the full Wechsler test of intelligence (Wechsler, 2003).

The *SRS* is a 65-item questionnaire (score 0–195), applied to the parents who report on various aspects of the child’s interpersonal communication, reciprocal behavior and repetitive/stereotypic behavior. The SRS identifies the presence and extent of autistic social impairments on a quantitative scale with higher scores indicating greater severity of social impairment (Constantino et al., 2003).

*Social Emotional Evaluation* is a task administered to the child. The SEE evaluates the social skills and higher level language skills that children need for successful interaction in everyday situations at home, at school and in the community. The SEE assesses both the receptive and expressive social skills of children aged 6–12, and includes tasks in which the child must identify common emotions, recognize emotional reactions, understand social gaffes, and understand conflicting messages. The following three scores are calculated: Receptive (0–59 points), Expressive (0–74 points), and Total score (0–133 points). A validation study reported in the SEE manual including children with ASD and TD children revealed large between-group effect sizes (Wigg, 2013).

*The SEE Social Emotional Questionnaire* (SEQ) is applied to the parents and contains 45 questions on the social skills of the child. The SEQ Raw score was divided by the total possible points (maximum 180 points with no excluded questions) after excluding questions with checkmarks in the ‘Don’t Know/Not Applicable’ column to obtain the Parent SEQ Total Percentage (Wigg, 2013).

### Statistical Analyses

Analyses were carried out using SPSS 20. Student’s *t*-tests and chi-square tests were applied to compare continuous and categorical background variables (gender, age in months, Wechsler Block Design scale score, and Wechsler Vocabulary scale score).

The test–retest reliability of the ToM-Frederik for the 11–12-year-old children in the TD group was established by means of a Pearson product-moment correlation coefficient. Furthermore, a paired samples *t*-test was applied to estimate the test–retest effect by comparing the ToM-Frederik Total score from the first assessment with the score from the second assessment.

Between-group comparisons were carried out to assess whether the outcome variable from the ToM-Frederik differed between the TD and the HFASD group. Not all children with HFASD completed all tasks (five missed one task; one missed two tasks; one missed three tasks or parts hereof). Thus, as we aimed to include as many children as possible in each between-group comparison, the number of participants varies. The between-group comparisons were done using ANCOVA, with the background variable(s) as the co-variate(s). When the raw data deviated significantly from a normal distribution, they were logarithmically (Strange Stories Physical State raw score; SEE Receptive score; SEE Total score) or square root transformed (AT MCQ-categorization raw score; SEE Expressive score; SRS raw score) to approximate a normal distribution before analyses were performed. The same procedure was followed for the outcome variables from Strange Stories, AT, SEE, and SRS.

To determine the convergent validity of ToM-Frederik, potential associations with the core variables derived from Strange Stories and AT were analyzed using Pearson correlations in the HFASD and TD groups separately. To assess the discriminant validity of the ToM-Frederik score, we assessed its potential associations with the two indices of intelligence (Wechsler Block Design scale score, and Wechsler Vocabulary scale score) in the subgroup that was assessed with these tests, using Pearson correlations.

Finally, the potential associations between ToM-Frederik and the SRS Total score were analyzed using Pearson correlations.

## Results

### Background Variables

There was a considerable, and statistically significant, larger percentage of boys in the HFASD compared to the TD group. Consequently all between-group analyses were adjusted for gender (see **Table 1**).

**Table 1:** Background variables.

	HFASD		TD		
	*N*	*M* (*SD*)	*N*	*M* (*SD*)	Analysis
Age in months	33	136.15 (23.04)	41	130.85 (20.81)	*t*(72) = 1.038, *p* = 0.303
Vocabulary	27	9.15 (3.13)	40	10.60 (3.15)	*t*(65) = -1.853, *p* = 0.068
Block Design	27	10.78 (2.72)	41	10.92 (4.14)	t(66) = -0.160, *p* = 0.874
FSIQ	14^∗^	103.43 (16.89)	0	-	–
	*N*	*N* (%)	*N*	*N* (%)	
Gender (*N*, % boys)	33	24 (73)	41	17 (41)	*R*^2^(1) = 7.233, *p* = 0.007

*HFASD, High Functioning Autism Spectrum Disorders; TD, typically developing; SD, standard deviation; FSIQ, Full Scale IQ. ^∗^Eight of the patients with ASD assessed with the two subtests also had a FSIQ score.*

### Test–Retest Reliability of ToM-Frederik

Ten out of the 14 participants aged 11–12 in the TD group, participated in the retest assessment of ToM-Frederik. The test-retest reliability estimate of the ToM-Frederik Total score was good (*r* = 0.84, *p* = 0.003). In addition, the test-retest effect of this score appeared relatively low and statistically non-significant (ToM-Frederik Total score: *M*_time1_ = 22.70, *SD* = 4.06) and *M*_time2_ = 24.30, *SD* = 2.58; *p* = 0.17).

### Between-Group Comparisons

#### Theory-of-Mind Tasks

ToM-Frederik Total raw score was significantly lower in the group of children with HFASD than in the TD group (see **Table 2**).

**Table 2:** Between-group comparisons.

	HFASD		TD		
	*N*	*M* (*SD*)	*N*	*M* (*SD*)	
ToM-Frederik	32	20.44 (4.9)	41	22.88 (4.4)	*F*(2,70) = 4.027, *p* = 0.049
**Strange stories**					
Mental state stories	31	8.45 (3.2)	41	10.90 (2.7)	*F*(2,69) = 10.407, *p* = 0.002
Physical state stories	31	8.16 (2.3)	41	9.83 (2.5)	*F*(2,69) = 7.854, *p* = 0.007
Mental state stories (adjusted for physical state stories)	31	8.45 (3.2)	41	10.90 (2.7)	*F*(2,69) = 6.810, *p* = 0.011
**Animated Triangles**					
**Subjective**					
Appropriateness					
Random	33	6.33 (3.9)	41	11.12 (1.4)	*F*(2,71) = 49.394, *p* < 0.0001
Goal-directed	33	7.00 (3.7)	41	10.37 (1.1)	*F*(2,71) = 26.433, *p* < 0.001
ToM	33	4.21 (2.3)	41	8.66 (1.7)	*F*(2,71) = 76.682, *p* < 0.001
Intentionality					
Random	26	1.03 (0.6)	41	0.47 (0.5)	*F*(2,64) = 14.663, *p* < 0.001
Goal-directed	28	2.27 (1.0)	41	2.42 (0.4)	*F*(2,66) = 1.151, *p* < 0.287
ToM	27	3.12 (0.6)	41	3.70 (0.4)	*F*(2,65) = 21.169, *p* < 0.001
Missing answers	33	2.79 (4.3)	41	0.02 (0.2)	*t*(72) = 4.099, *p* < 0.001
**Objective**					
MCQ-categorization	33	8.55 (2.7)	41	9.88 (1.5)	*F*(2,71) = 8.249, *p* < 0.005
MCQ-feelings	33	3.30 (2.2)	41	4.24 (2.1)	*F*(2,71) = 3.403, *p* < 0.069
**The Social Emotional Evaluation (SEE)**					
Receptive score	32	52.19 (4.1)	41	54.09 (2.9)	*F*(2,70) = 3.080, *p* = 0.084
Expressive score	32	37.69 (10.5)	41	40.02 (6.6)	*F*(2,70) = 1.102, *p* = 0.297
Total score	32	89.88 (13.3)	41	94.12 (9.0)	*F*(2,70) = 1.736, *p* = 0.192
**The SEE Social Emotional Questionnaire (SEQ)**					
Percent of maximum points	33	0.57 (0.2)	41	0.87 (0.2)	*F*(2,71) = 32.679, *p* < 0.001
Social Responsiveness Scale (SRS)	33	85.52 (30.0)	41	19.76 (13.2)	*F*(2,71) = 171.359, *p* < 0.001

Both the Strange Stories Mental State raw score and the Strange Stories Physical State (logarithmically transformed) raw score were significantly lower in the group of children with HFASD than in the TD group. Still, the Mental State raw score remained significantly lower in the HFASD group than in the group of children when adjusted for Physical State raw score (see **Table 2**).

With regard to the AT the score for AT Appropriateness was significantly lower for the HFASD than for the TD group in the Random, Goal-Directed, and ToM-type of animations. The AT Intentionality score for the ToM-type animations was significantly lower in the HFASD group than the TD group. Conversely, for Random-type animations, the intentionality score was significantly higher in the HFASD group than in the TD group. For the Goal-directed-type animations, there was no significant difference between the HFASD group and the TD group (see **Table 2**). The AT MCQ-categorization (square root transformed) Total score was significantly lower in the HFASD group than in the TD group. No significant difference was observed between the groups for the AT MCQ-feelings Total score, though a statistical trend was observed. A *post hoc* analysis revealed the participants in the HFASD group had significantly more AT missing answers than the participants in the TD group (see **Table 2**).

#### Social Emotional Evaluation

The HFASD and the TD group did not differ with respect to the SEE Receptive (logarithmically transformed) score, the SEE Expressive (square root transformed) score or the SEE Total (logarithmically transformed) score. For the SEE Social Emotional Questionnaire (SEQ), a significantly lower percentage of the maximum possible result was awarded to the HFASD than the TD group. As SEE is only aimed at children aged 6–12, these analyses where repeated without the children aged 13–14. However, this did not significantly change the results (data not shown). Thus, as SEE did not differentiate between the groups, it was not included in the subsequent analyses.

#### Social Responsiveness Scale

The HFASD group had significantly higher SRS (square root transformed) Total score than the TD group, indicating greater severity of social impairment for the HFASD group.

#### Convergent and Discriminant Validity

As shown in **Table 3**, the score on ToM-Frederik was significantly and positively correlated with both the Strange Stories Mental State score and the AT ToM intentionality score in the HFASD group. Furthermore, the Strange Stories Mental State score and the AT ToM intentionality score were significantly and positively correlated with each other. However, while the score on ToM-Frederik was significantly and positively correlated with the Strange Stories Mental State score in the TD group, it was not significantly correlated to the AT ToM intentionality score. Furthermore the Strange Stories Mental State score and the AT ToM intentionality score did not correlate significantly (see **Table 3**).

**Table 3:** Correlations between measures of Theory-of-Mind.

	Strange Stories			AT		
	*r*	*p*	*N*	*r*	*p*	*N*
**HFASD**						
ToM-Frederik	0.570	0.001	31	0.484	0.011	27
Strange stories	–	–	–	0.521	0.005	27
**TD**						
ToM-Frederik	0.462	0.002	41	0.125	0.434	41
Strange stories	–	–	–	0.117	0.468	41

*HFASD, High Functioning Autism Spectrum Disorders; TD, typically developing; SRS, Social Responsiveness Scale.*

Analyses showed that ToM-Frederik was not significantly correlated with SRS in neither the HFASD nor the TD group (see **Table 4**).

**Table 4:** Correlations between Social Responsiveness Scale and measures of Theory-of-Mind.

	SRS		
	*r*	*p*	*N*
**HFASD**			
ToM-Frederik Total score	-0.061	0.738	32
Strange Stories Mental State score	-0.084	0.653	31
Animated Triangles ToM Intentionality score	-0.137	0.495	27
**TD**			
ToM-Frederik Total score	-0.100	0.532	41
Strange Stories Mental State score	-0.200	0.009	41
Animated Triangles ToM Intentionality score	-0.121	0.434	41

*HFASD, High Functioning Autism Spectrum Disorders; TD, typically developing; SRS, Social Responsiveness Scale.*

In terms of discriminant validity of the ToM-Frederik task, we observed no significant associations between the indices of intelligence and the ToM-Frederik score in neither the full sample (Vocabulary: *r* = 0.035, *p* = 0.779, *n* = 67; Block Design: *r* = -0.026, *p* = 0.834, *n* = 66) nor the TD group (Vocabulary: *r* = -0.127, *p* = 0.433, *n* = 40; Block Design: *r* = -0.100, *p* = 0.545, *n* = 39) or the HFASD group (Vocabulary: *r* = 0.132, *p* = 0.513, *n* = 27; Block Design: *r* = 0.098, *p* = 0.628, *n* = 27).

## Discussion

The main aim of the present study was to examine aspects of validity and reliability of the Danish versions of the ToM-Frederik as a measure of ToM deficits. Our findings support the validity of ToM-Frederik as a measure of ToM as it was able to identify, at the group level, the expected ToM deficits in this sample of older children and young adolescents with HFASD. This is in line with previous findings in a study comparing 3–12 year-old children with PDD-NOS and TD children using the Dutch version of ToM-Frederik (Blijd-Hoogewys et al., 2008). Furthermore, the convergent validity of ToM-Frederik as a measure of ToM was supported by its significant positive associations with the Strange Stories and the AT scores in the HFASD group, whereas ToM-Frederik was only significantly correlated with Strange Stories, but not with AT, in the TD group. Based on the current data it is not possible to give conclusive explanations of this latter and unexpected finding. However, it might be caused by the relatively low variation in AT Intentionality scores. The discriminant validity of ToM-Frederik as a measure of TOM was supported by its non-significant associations with our indices of intelligence; this was the case for the full sample as for the TD and HFASD groups.

ToM-Frederik was not significantly associated with SRS in neither the HFASD nor the TD group. The lack of a significant association in the HFASD group may be explained by the repetitive/stereotypic behavior items included in the SRS. The repetitive/stereotypic behaviors of ASD may be associated with executive function deficits, whereas ToM-impairments in ASD may be associated more with impairments of reciprocal interaction and communication (Hughes et al., 1994; Joseph, 1999). The SRS covers all three dimensions of impairments in ASD (reciprocal interaction, communication, and repetitive/stereotypic behaviors). Therefore the items on repetitive/stereotypic behavior may have confounded the potential associations between real life communication and social interaction deficits and ToM.

The findings are also in support of the Danish versions of the Strange Stories and AT as measures of ToM, because they were able to identify, at the group level, the expected ToM deficits in this sample of older children and young adolescents with HFASD. The findings on the Strange Stories task are in line with previous findings reporting significant group differences in the Mental State stories in a study comparing 7–12-year-old children with autism and TD children (White et al., 2009). However, in contrast to the latter study, we also found significant group differences in the Physical State stories. Nevertheless, when adjusted for Physical State stories the group differences for the Mental State stories remained significant. Thus, the difference in score seems to reflect a specific impairment of ToM.

In terms of AT, the current findings are in line with previous work in children and adolescents reporting there are significant differences between groups of HFASD and TD (Salter et al., 2008; Schwenck et al., 2012). Only one of these studies did assess AT Intentionality, but in contrast to the current study, Salter et al. (2008) did not find significant between-group differences in the AT Intentionality score for any type of animations in their 6–20-year old sample. In terms of the objective AT scoring, we only found a significant lower MCQ categorization score in the children and adolescents with HFASD, whereas White et al. (2011) also observed a significant difference for the MCQ feelings in adults. This objective AT scoring measure has not previously been applied to children and young adolescents. As only about 50% of the answers in the TD group were correct, the MCQ Feelings part of the task may be too difficult for this young age group.

The findings are in support of the SRS; the biggest gap in ToM scores between HFASD and TD was found on this test. In addition, our findings support validity of the SEQ parent questionnaire as a sensitive measure of real life social and communicative behavior deficits in children and adolescents with HFASD. On the other hand, the SEE task was not supported as a valid measure of impairments of social skills, as the scores did not differ significantly between the groups on any SEE scales. In terms of the SEE Receptive, both groups score close to ceiling which may indicate that this part of the test is too easy for many children with TD and even for many children with HFASD. In contrast, it may be too difficult for the children with TD to attain high scores on the SEE Expressive. However, based on the present dataset, it is difficult to provide any conclusive explanations of the lack of significant between-group differences.

### Strengths and Limitations

The main strength of this study is the relatively comprehensive social cognitive test battery, making us able to assess convergent validity of the TOM-Frederik Task. Seven of the 33 children in the HFASD group did not complete all tasks. These children may have a lower level of functioning, but as we do not have comparable IQ data for all participants it is not possible to assess this in the present dataset. However, all participants have an IQ within the normal range.

The present study also has some limitations. The gender distribution differed between the HFASD and the TD group, with more boys in the HFASD group. A number of previous studies have reported significant gender differences in ToM (Charman, 2002; Baron-Cohen et al., 2003; Sabbagh et al., 2006), and although not all studies find significant gender differences on ToM development (Calero et al., 2013), gender may be a confounding factor.

The assessment of the test–retest-reliability of the ToM-Frederik task was not based on an age representative subsample and only included a small subsample of 11–12-year-olds from the TD group. Consequently, the test–retest-reliability estimate may not be generalizable to other age groups of children with TD or to children with HFASD. The test–retest reliability of the Danish version of ToM-Frederik was not assessed for children with HFASD. A previous study has found good test–retest-reliability of the original ToM-Frank in children with pervasive developmental disorder not otherwise specified (Blijd-Hoogewys et al., 2008).

### Directions for Future Research

Future research on ToM-Frederik should include groups with other psychiatric diagnoses in order to assess trans-diagnostic applicability of the tests. Furthermore, future research should also include more participants across the age-span in order to be able to identify at what age AT MCQ Feelings becomes able to identify significant group differences. Finally, the separation of the two groups by ToM-Frederik may be due to other differences picked up by the instruments, e.g., differences in language and motivation. Future studies should further explore to what degree the performance on the ToM-Frederik is influenced by such factors.

## Conclusion

The current study provides overall support for the validity of the Danish version of the Theory-of-Mind Storybook Frederik as a measure of ToM. The task was able to identify, at the group level, the expected ToM deficits in this sample of older children and young adolescents with HFASD. Furthermore, the convergent validity of ToM-Frederik as a measure of ToM was supported by significant positive associations with the Strange Stories and the AT scores in the HFASD group, whereas ToM-Frederik was significantly correlated with Strange Stories, but not with AT, in the TD group. Finally, ToM-Frederik was not significantly associated with SRS in neither the HFASD nor the TD group. The task represents a gain for both research and clinical practice, as it is relatively fast to administer and provides outcome based on a highly nuanced scoring. All three ToM tasks were able to discriminate between the two groups but the scoring system of the ToM-Frederik allows for a more detailed categorization of different types of ToM deficits.

## Author Contributions

LC, AB-V, RJ, JvO, EB-H, LA, MV, PD, MD, PJ and JJ. LC, PJ, and JJ designed the study. RJ and LA made Substantial contributions to the acquisition of the data. LC, AB-V, JvO, EB-H, MV, PD, MD, PJ, and JJ made substantial contributions to the analysis and interpretation of data for the work. LC made the draft and all authors contributed to revising it critically for important intellectual content. All authors gave a final approval of the version to be published. All authors agreed to be accountable for all aspects of the work in ensuring that questions related to the accuracy or integrity of any part of the work are appropriately investigated and resolved.

## Conflict of Interest Statement

The authors declare that the research was conducted in the absence of any commercial or financial relationships that could be construed as a potential conflict of interest.
